# Design and proof of concept for targeted phage-based COVID-19 vaccination strategies with a streamlined cold-free supply chain

**DOI:** 10.1073/pnas.2105739118

**Published:** 2021-07-07

**Authors:** Daniela I. Staquicini, Fenny H. F. Tang, Christopher Markosian, Virginia J. Yao, Fernanda I. Staquicini, Esteban Dodero-Rojas, Vinícius G. Contessoto, Deodate Davis, Paul O’Brien, Nazia Habib, Tracey L. Smith, Natalie Bruiners, Richard L. Sidman, Maria L. Gennaro, Edmund C. Lattime, Steven K. Libutti, Paul C. Whitford, Stephen K. Burley, José N. Onuchic, Wadih Arap, Renata Pasqualini

**Affiliations:** ^a^Rutgers Cancer Institute of New Jersey, Newark, NJ 07101;; ^b^Division of Cancer Biology, Department of Radiation Oncology, Rutgers New Jersey Medical School, Newark, NJ 07103;; ^c^Center for Theoretical Biological Physics, Rice University, Houston, TX 77005;; ^d^Department of Physics, Institute of Biosciences, Humanities and Exact Sciences, São Paulo State University, São José do Rio Preto, SP 15054, Brazil;; ^e^Public Health Research Institute, Rutgers New Jersey Medical School, Newark, NJ 07103;; ^f^Department of Neurology, Harvard Medical School, Boston, MA 02115;; ^g^Rutgers Cancer Institute of New Jersey, New Brunswick, NJ 08901;; ^h^Department of Surgery, Rutgers Robert Wood Johnson Medical School, New Brunswick, NJ 08901;; ^i^Department of Physics and Center for Theoretical Biological Physics, Northeastern University, Boston, MA 02115;; ^j^RCSB Protein Data Bank and Institute for Quantitative Biomedicine, Rutgers, The State University of New Jersey, Piscataway, NJ 08854;; ^k^Department of Chemistry and Chemical Biology, Rutgers, The State University of New Jersey, Piscataway, NJ 08854;; ^l^RCSB Protein Data Bank, San Diego Supercomputer Center and Skaggs School of Pharmacy & Pharmaceutical Sciences, University of California San Diego, La Jolla, CA 92067;; ^m^Department of Biosciences, Rice University, Houston, TX 77005;; ^n^Department of Chemistry, Rice University, Houston, TX 77005;; ^o^Department of Physics and Astronomy, Rice University, Houston, TX 77005;; ^p^Division of Hematology/Oncology, Department of Medicine, Rutgers New Jersey Medical School, Newark, NJ 07103

**Keywords:** AAVP, COVID-19, gene delivery, phage display, SARS-CoV-2

## Abstract

Development of effective vaccines against coronavirus disease 2019 (COVID-19) is a global imperative. Rapid immunization of the entire human population against a widespread, continually evolving, and highly pathogenic virus is an unprecedented challenge, and different vaccine approaches are being pursued. Engineered filamentous bacteriophage (phage) particles have unique potential in vaccine development due to their inherent immunogenicity, genetic plasticity, stability, cost-effectiveness for large-scale production, and proven safety profile in humans. Herein we report the development and initial evaluation of two targeted phage-based vaccination approaches against SARS-CoV-2: dual ligand peptide-targeted phage and adeno-associated virus/phage (AAVP) particles. For peptide-targeted phage, we performed structure-guided antigen design to select six solvent-exposed epitopes of the SARS-CoV-2 spike (S) protein. One of these epitopes displayed on the major capsid protein pVIII of phage induced a specific and sustained humoral response when injected in mice. These phage were further engineered to simultaneously display the peptide CAKSMGDIVC on the minor capsid protein pIII to enable their transport from the lung epithelium into the systemic circulation. Aerosolization of these “dual-display” phage into the lungs of mice generated a systemic and specific antibody response. In the second approach, targeted AAVP particles were engineered to deliver the entire S protein gene under the control of a constitutive CMV promoter. This induced tissue-specific transgene expression, stimulating a systemic S protein-specific antibody response in mice. With these proof-of-concept preclinical experiments, we show that both targeted phage- and AAVP-based particles serve as robust yet versatile platforms that can promptly yield COVID-19 vaccine prototypes for translational development.

## Significance

The COVID-19 pandemic has had an unprecedented impact. Although several vaccines have received emergency use authorization, demand has created enormous logistical challenges—including supply, access, and distribution—that justify research for alternative strategies. Phage are viruses that only infect bacteria and can be safely administered to humans. Here, as a proof-of-concept study, we demonstrate that aerosol vaccination with lung-targeted phage particles displaying short SARS-CoV-2 S protein epitopes and subcutaneous vaccination with targeted AAVP particles carrying the entire S protein gene both elicit systemic and specific immune responses in immunocompetent mice. Given their unique attributes, including sturdiness, simple-to-engineer platform, cost-effectiveness for rapid large-scale production, and stability at room temperature, these phage-based approaches may become attractive tools for COVID-19 vaccine development.

Since early 2021, the World Health Organization (WHO) has estimated that nearly 3 million deaths in 223 countries/territories have been caused by complications of coronavirus disease 2019 (COVID-19). This unprecedented pandemic has prompted a worldwide collaborative effort to develop vaccines and antiviral therapies to control global spread. Severe acute respiratory syndrome coronavirus-2 (SARS-CoV-2) is the third zoonotic coronavirus to infect humans in less than 20 y (1, 2). Previous coronavirus epidemics, such as severe acute respiratory syndrome (SARS) and Middle East respiratory syndrome (MERS), foreshadowed the risk of emerging disease outbreaks and the imminent need for novel and versatile technologies for rapid manufacturing and large-scale distribution of vaccines and therapies as emergency countermeasures.

SARS-CoV-2 is a single-stranded enveloped RNA virus with four main structural proteins. The spike (S) protein mediates both host cell recognition and membrane fusion and is pivotal for viral entry. The S protein, composed of the S1 and S2 subunits, is displayed as a trimer on the surface of the viral particle (3). Within the S1 subunit, the receptor-binding domain (RBD) adopts an open conformation that interacts with the angiotensin-converting enzyme 2 (ACE2) receptor on the host cell membrane. Upon binding, the S1 subunit is cleaved and subsequent conformational changes in the S2 subunit trigger the formation of a six-helical bundle composed of heptapeptide repeat sequence-1 (HR1) and heptapeptide repeat sequence-2 (HR2), followed by the insertion of the fusion peptide (FP) into the host cell membrane. Given the importance of the S protein for the entry of SARS-CoV-2 in the host cells, it has served as the main target for vaccines and therapeutic antibodies. Thus, the identification and understanding of structurally defined S protein epitopes with potential neutralizing capabilities are crucial in the design of effective and robust vaccines and/or therapeutic antibody mixtures (4, 5).

Current vaccine-design and -development platforms against COVID-19 are broadly classified into categories that include nucleic acid-based vaccines (mRNA or DNA), viral vector vaccines (e.g., adenovirus), inactivated or live attenuated viral vaccines, or recombinant protein- or peptide-based vaccines. Some have been granted emergency use authorization (EUA) by various regulatory agencies, including two mRNA vaccines (Pfizer-BioNTech and Moderna), three nonreplicating adenovirus vaccines (Oxford/AstraZeneca, Sputnik V, and Johnson & Johnson), and an inactivated SARS-CoV-2 vaccine (CoronaVac), while several others are pending formal approval and/or undergoing clinical trials. In addition to such extraordinary global efforts, further research and development of vaccines that might be amenable to temperature fluctuation, rapid large-scale production and distribution, and conferral of long-term immunological protection in the face of existing and emerging viral variants remain an unmet need (6, 7).

Phage particles have been used in medical settings for nearly a century and represent an inexpensive and versatile tool for large-scale immunization. Lytic phage particles are viruses that naturally infect bacteria; during the preantibiotic era, humans received phage particles to neutralize systemic bacterial infections without severe adverse effects (8, 9). Recently, engineered phage particles have been leveraged in different translational applications, and particularly in vaccine development because they are: 1) easy to genetically engineer and produce in bacterial hosts in large quantities; 2) strong immunogens capable of stimulating both cellular and humoral immunity (10–12); and 3) stable under harsh environmental conditions (pH and temperature). Such biological attributes facilitate generation, transport, storage, and administration (10); and most notably 4) they are generally considered safe for administration in humans (9, 13–15). We and others have shown that the addition of targeting peptide sequences to the minor coat protein III (pIII) and immunogenic peptide sequences on the recombinant protein VIII coat protein (rpVIII) confers tropism of targeted phage particles to specific mammalian cell surface receptors or even to subcellular organelles in normal or diseased tissues (16–21). Pertinent to vaccine design and development, we have recently demonstrated that aerosolized lung-targeted phage particles subsequently undergo ligand/receptor-mediated transport into the systemic circulation, are safe, and elicit specific and sustained local and systemic immune responses in mice and nonhuman primates (22, 23). Further genome engineering served to create a hybrid adeno-associated virus/phage (AAVP) vector (20), which has been extensively validated for ligand-directed gene delivery and represents a viable alternative for nucleotide-based vaccines (mRNA or DNA). A few favorable features were built into the vector design, namely: 1) the adjuvant immune properties of phage particles, 2) a ligand-directed system for ligand/receptor-mediated internalization, 3) a well-characterized fate of the genome post vector internalization (e.g., head-to-tail concatemerization and intact genome integration), and 4) ability to avoid neutralization after an immune response against phage, as demonstrated in repeat-dose studies in several animal models (20, 24–28), including pet dogs with native tumors (29).

Therefore, we explored the inherent biological and genetic properties of targeted phage and AAVP particles to produce vaccine candidates against COVID-19. First, guided by structure-based antigen design, we selected S protein epitopes that were genetically incorporated into the rpVIII of the f88-4 phage genome. The ligand peptide CAKSMGDIVC, which has just been reported for its unique ability to target lung epithelium cells and induce the transport of targeted phage particles into systemic circulation (22, 23), was then cloned into the pIII of the fUSE55 viral genome, creating a dual-display system with potential for efficient delivery and immunization against the SARS-CoV-2 S protein. This approach allows us to use the lung as the main route of immunization, which can have significant advantages over conventional administration methods. Aerosol administration is needle-free and thereby minimizes the requirement for specialized medical staff. In addition, it is not subject to first-pass metabolism, and is considered the most effective route for inducing local immune protection against airborne pathogens (30–33). In a second approach, we used the AAVP vector (20), which contains a eukaryotic expression cassette flanked by the inverted terminal repeat (ITR) sequences of AAV within the phage genome, for the delivery and transduction of the full-length S protein gene (AAVP *S*) in host cells. This alternative approach leverages a similar rationale that serves as the basis for the activity of existing vaccines (either mRNA or adenovirus), which triggers a specific humoral response against the full-length S protein. Integration of large DNA antigen sequences flanking the AAV transgene of the AAVP confers stability to the construct upon transduction and enhanced expression efficiency. Both phage- and AAVP-based vaccines elicited a systemic S protein-specific humoral response in mice with no evidence of adverse effects, indicating that both technologies might hold promise in vaccine development.

## Results

### Development of Phage- and AAVP-Based Vaccine Platforms against SARS-CoV-2.

**Fig. 1.**
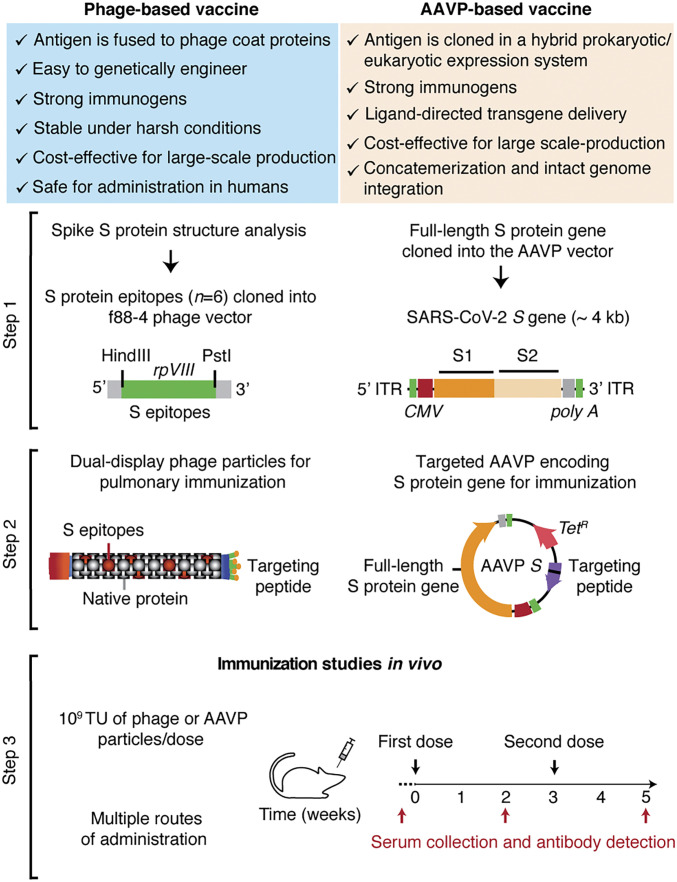
Representation of the phage- and AAVP-based vaccine candidates. Scheme of the approach used for the conception, design, and application of two strategies for immunization against the SARS-CoV-2 S protein using phage particles. Step 1: Structural analysis, selection of structurally defined epitopes and cloning steps for the generation of dual-display phage particles and AAVP encoding the full-length S protein. Step 2: Molecular engineering of single- and dual-display phage particles, and AAVP *S* constructs. Step 3: Functional validation and immunization studies in vivo in mice.

We pursued two different strategies for immunization: 1) phage-based vaccine candidates displaying selected S protein epitopes, and 2) an AAVP-based vaccine candidate that utilizes the entire SARS-CoV-2 S protein (Fig. 1). In each approach, we incorporated a ligand peptide along with the viral antigens in the phage or AAVP to target specific cell surface receptors and facilitate an immune response.

For the expression of antigens in the capsid system, we genetically engineered phage particles to display immunologically relevant S protein epitopes (see below) on the highly exposed rpVIII protein of the phage capsid by using the f88-4 vector (Fig. 1, step 1) (18, 19). To enable tissue-specific targeting of these phage particles, we also subcloned the coding sequence of the ligand CAKSMGDIVC peptide into the pIII gene of the fUSE55 vector, yielding a dual-display phage (Fig. 1, step 2). The ligand CAKSMGDIVC binds specifically to α_3_β_1_ integrins and thereby mediates the transport of targeted phage particles across the lung epithelium into systemic circulation where they elicit strong and sustained pulmonary and systemic humoral responses against antigens displayed on the phage capsid (22, 23). As a negative control, we used the untargeted parental phage particles (insertless phage), which display the native pIII and pVIII viral proteins.

For our second strategy, based on gene delivery, we inserted an expression cassette containing the full-length S protein transgene and the human cytomegalovirus (*CMV*) promoter in a *cis* conformation within the 5′ and 3′ ITR elements within the targeted AAVP genome for gene delivery and transduction into host cells (Fig. 1, step 1). As a negative control, we used the targeted AAVP empty vector (termed “AAVP *S-null*”). Targeting was established by the display of a double-cyclic α_v_ integrin-binding peptide, CDCRGDCFC (RGD-4C), as a ligand on pIII (Fig. 1, step 2). This peptide has high-affinity for α_v_β_3_ and/or α_v_β_5_ integrins (17, 34), which are highly expressed in trafficking leukocytes to draining lymph nodes and areas of inflammation (35). The classic arginine-glycine-aspartate (Arg-Gly-Asp, RGD) tripeptide motif is known to facilitate particle uptake by dendritic cells and enhance the immunogenicity of peptide antigens, DNA vaccines, and adenovirus vectors (36–38).

Both approaches allow for rapid molecular swapping of targeting motifs, epitopes, and/or gene-encoding sequences through conventional recombinant genetics. This enables rapid and powerful design flexibility to produce a variety of vaccines that can overcome potential limitations in preserving protein conformation of structure-based epitopes. The dual-display phage particles, the RGD-4C–targeted AAVP *S* particles, plus the corresponding controls, were tested in vivo in immunocompetent mice to assess the different routes of administration and to evaluate the induced antigen-specific humoral response by ELISA (Fig. 1, step 3). The overall immunization schedule included at least two administered doses of 10^9^ transducing units (TU) of phage- or AAVP-based particles with an interval of 1 to 2 wk, as indicated.

### Identification and Selection of Epitopes for Dual-Display Phage-Based Vaccine.

To identify relevant epitopes for phage or AAVP capsid manipulation, in silico analysis of the experimentally determined viral S protein structure of the Wuhan-Hu-1 strain (GenBank accession no. NC_045512.2) was performed. We prioritized solvent-exposed amino acid residue stretches with flanking cysteine residues and cyclic conformations because such sequences might be more likely to recapitulate the composition of endogenous epitopes and could thereby increase antigen recognition and processing by the host immune system. Other epitopes were also considered following structure-guided principles, even in the absence of flanking cysteine residues. Also, given that targeted phage particles are produced in prokaryotic organisms, we prioritized epitopes lacking sites expected to undergo posttranslational modification.

**Fig. 2.**
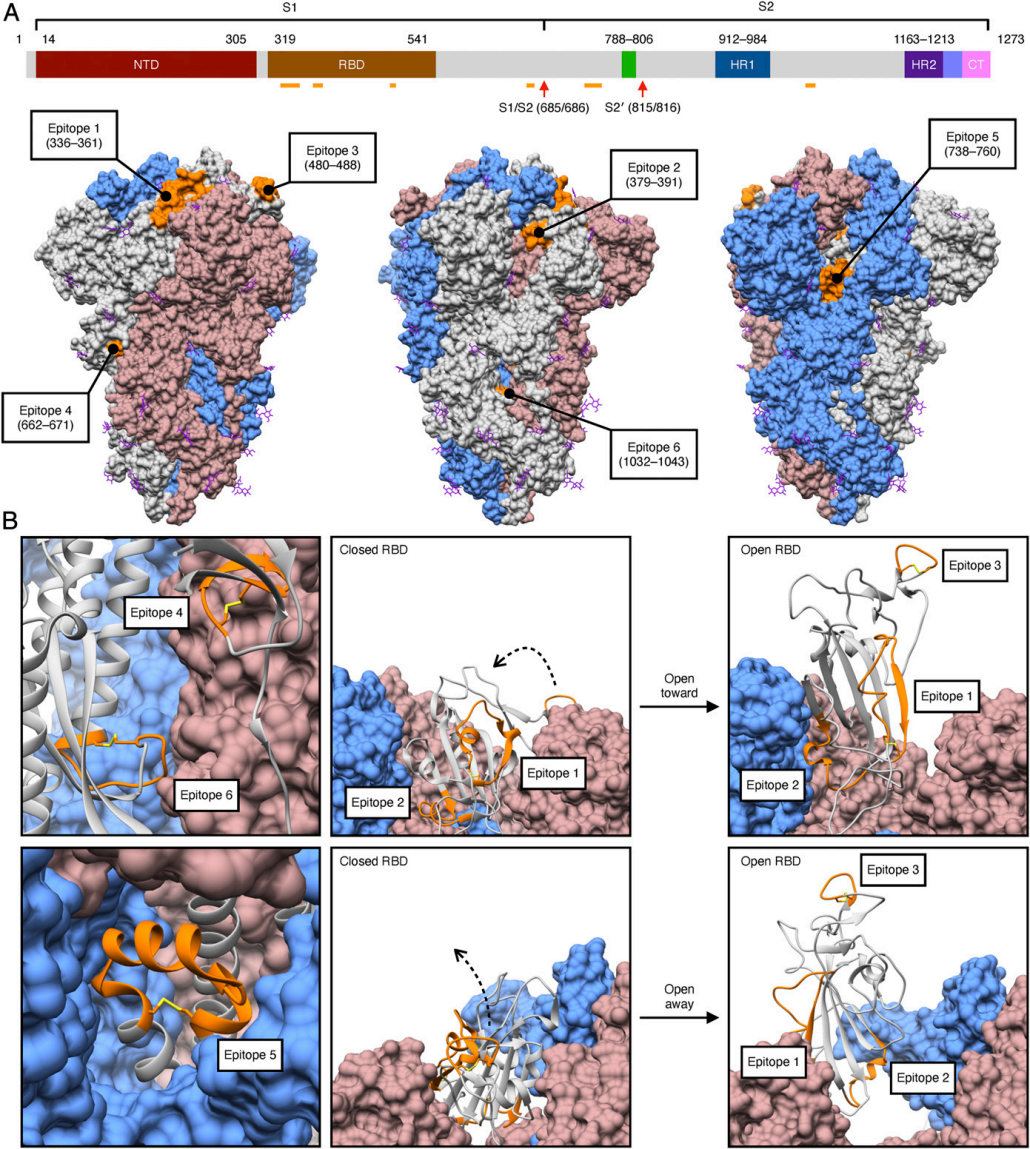
Identification of structural epitopes on the S protein trimer. (*A*) Six epitopes (orange) spanning the SARS-CoV-2 S protein were selected for display on the rpVIII protein. Four epitopes are located within the S1 subunit, epitope 1 (aa 336 to 361), epitope 2 (aa 379 to 391), epitope 3 (aa 480 to 488), epitope 4 (aa 662 to 671), and two within the S2 subunit, epitope 5 (aa 738 to 760), and epitope 6 (aa 1,032 to 1,043). These epitopes are solvent exposed in the surface representation of the predominantly closed-state conformation of the S protein trimer (gray, cornflower blue, and rosy brown) (PDB ID: 6ZP0) (63). Glycans (shown in purple) are present throughout the structure; of the six epitopes, only epitope 1 (aa 336 to 361) contains a site for glycosylation (at N343). Two cleavage sites located in the S2 subunit, S1/S2 (aa 685/686) and S2′ (aa 815/816), are represented by red arrows. (*B*) All of the epitopes (orange) maintain a cyclic conformation in the ribbon representation of a S protein protomer (gray); disulfide bridges (yellow) are present between the flanking cysteine residues of all epitopes except on epitope 2 (aa 379 to 391). The open-state conformation of the S protein trimer with one RBD erect displays a change in orientation of epitopes 1, 2, and 3, though all remain solvent exposed (PDB ID: 6ZGG) (64). NTD, N-terminal domain; RBD, receptor-binding domain; HR1 and HR2, heptapeptide repeat sequence-1 and -2; CT, C terminus.

We selected six S protein epitopes, which are accessible in both the closed and open states of the S protein (Fig. 2). At least five of these epitopes have since been shown to be fully or partially immunogenic (*SI Appendix*, Fig. S1 and Table S1) (39–46). The six epitopes range in length from 9 to 26 amino acid (aa) residues. Four occur within the S1 subunit and two are found in the S2 subunit (Fig. 2*A*). Three of the S1 epitopes are located in the RBD: epitope 1 (aa 336 to 361), epitope 2 (aa 379 to 391), and epitope 3 (aa 480 to 488). The remaining epitope derived from the S1 subunit, epitope 4 (aa 662 to 671), is located near the cleavage site between the S1 and S2 subunits. Epitopes within the S2 subunit, epitope 5 (aa 738 to 760) and epitope 6 (aa 1,032 to 1,043), are located near the FP (aa 788 to 806) and HR1 (aa 912 to 984), respectively. Most of the selected epitopes are cyclic due to the presence of flanking cysteine residues, except epitope 2 (aa 379 to 391), which adopts a loop-like conformation despite the absence of a disulfide (Cys–Cys) bridge (Fig. 2*B*).

Many studies have demonstrated that the S protein is highly glycosylated, and some glycosylation sites have been reported to alter the infectivity of variants and facilitate evasion of the host immune response (4, 7). Since immune recognition relies on the structural conformation of the epitopes, much attention is needed towards glycosylation. For example, we identified that epitope 1 contains a glycosylation site on residue N343. Notably, this site seems to be important in viral infectivity as the glycosylation deletion N343Q markedly reduces infectivity of the D614G variant (7). In our system, however, we expect that the lack of *N*-glycosylation will not produce a substantial structural divergence in the epitope conformation when displayed on the phage capsid, as similarly observed with other SARS-CoV strains (47). Because the glycosylation site of epitope 1 is located in its N terminus, unlikely to interrupt antibody recognition of the remaining structure, we sought to investigate the efficacy of this additional epitope.

### Epitope Conformational Analysis by Molecular Dynamics.

**Fig. 3.**
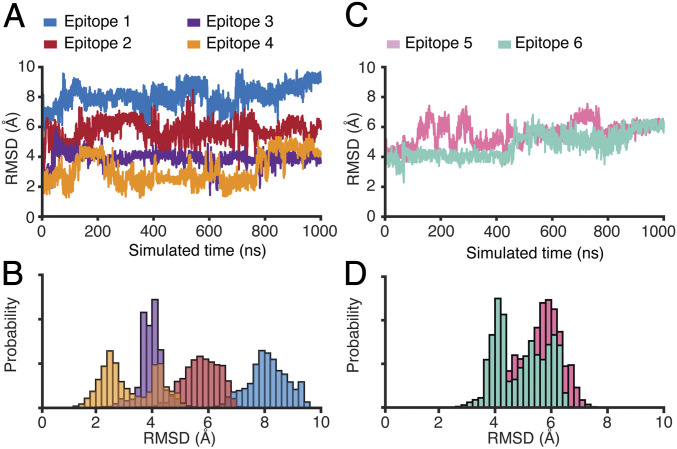
Explicit-solvent simulations reveal the near-native conformation of epitope 4 relative to the full-length S protein. (*A*) rmsd from the S protein conformation for each epitope within the S1 subunit: epitope 1 (aa 336 to 361), epitope 2 (aa 379 to 391), epitope 3 (aa 480 to 488), and epitope 4 (aa 662 to 671). Epitope 4 frequently samples low values (∼2 Å), while the other epitopes undergo substantial conformational rearrangements (rmsd >4 Å). (*B*) Probability as a function of rmsd shows that epitope 4 is most likely to sample conformations that are similar (rmsd ∼2.5 Å) to the S protein conformation. (*C*) rmsd as a function of time for the epitopes within the S2 subunit: epitope 5 (aa 738 to 760) and epitope 6 (aa 1,032 to 1,043). Both epitopes rapidly adopt and maintain large rmsd values (*D*).

To examine whether the predicted conformation of each epitope displayed on the phage or AAVP capsid would recapitulate its features on the S protein, we performed in silico conformational analysis through the use of microsecond-scale all-atom explicit-solvent simulations of each epitope. We hypothesized that epitope conformation is a prerequisite for successful induction of the immune response against the native S protein. Notably, we have found that, of the four S1 subunit epitopes considered, epitope 4 has the lowest spatial rmsd from the S protein conformation in two sets of simulations (Fig. 3 and *SI Appendix*, Fig. S3). While its rmsd fluctuates between 2 Å and 4 Å (Fig. 3*A*), epitope 4 has the most frequent low values (rmsd ∼2 Å), consistent with its adopting a near-native conformation when compared to the structure of the S protein (Fig. 3*B*) (48). As for the other selected epitopes of the S1 subunit (epitopes 1, 2, and 3) and the S2 subunit (epitopes 5 and 6), rmsd values are substantially higher (4 to 10 Å) (Fig. 3 *C* and *D*) after an initial relaxation of each system (∼100 ns). These differential rmsd trends suggest that epitope 4 is most likely to resemble the native conformation and generate IgG antibodies with high cross-reactivity with the S protein. If true, epitope 4 would represent a promising candidate for display on phage capsids that could elicit an immune response toward an exposed region of the S protein. It would also demonstrate that molecular dynamics simulations could serve as a tool for prioritizing epitopes with higher likelihood of recapitulating native conformation when selecting epitope candidates for use in targeted phage- or AAVP-based vaccines.

### Immunogenicity of Structurally Defined S Epitopes in Mice.

To evaluate the immunological potential of each S epitope and select a promising candidate(s) for phage- and AAVP-based vaccine development, we evaluated their ability to induce an immune response in immunocompetent mice. Six f88-4 phage were produced, each exhibiting one of the S protein epitopes (*n* = 6) fused into the rpVIII protein on ∼300 to 500 copies per phage particle (termed “single-display phage”) (*SI Appendix*, Fig. S2).

**Fig. 4.**
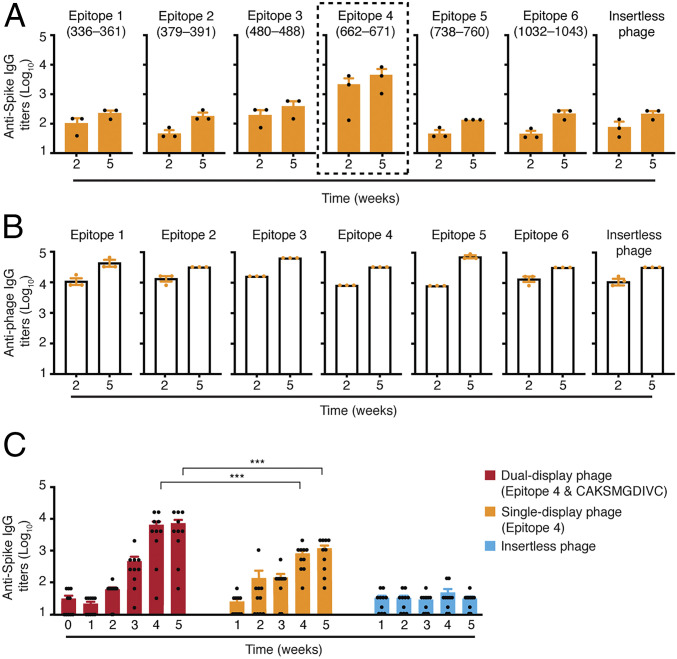
Immunogenicity of S protein epitopes on single-display and dual-phage particles. Five-week-old female Swiss Webster mice were immunized via SC administration of single-display phage constructs containing one of the six different epitopes expressed on rpVIII protein or the negative control insertless phage. Animals received a boost injection 3 wk after the first administration. (*A*) S protein-specific IgG antibodies and (*B*) phage-specific IgG antibodies were evaluated in sera of mice after 2 and 5 wk by ELISA (*n* = 3 mice per group). (*C*) Five-week-old female BALB/c mice were immunized via IT administration with epitope 4/CAKSMGDIVC dual-display phage particles, epitope 4 single-display phage particles, or the control insertless phage. Animals received a boost 3 wk after the first administration. S protein-specific IgG antibodies were evaluated weekly by ELISA (*n* = 10 mice per group). Data represent ± SEM (***P < 0.001).

Immunogenicity of the epitopes expressed on the rpVIII protein was assessed in mouse serum samples (from either Swiss Webster or BALB/c, as indicated) obtained after the first (prime) dose and the second (booster) dose administered subcutaneously (SC) and compared against the negative control (insertless phage particles). Antigen-specific IgG titers were quantified by ELISA by using an immobilized recombinant S protein (aa 16 to 1,213) for capture. Epitope 4 from the S1 subunit induced high titers of S protein-specific IgG antibodies, and booster immunizations further increased antibody levels (Fig. 4*A*) relative to the other five targeted constructs and the negative control insertless phage. These results indicate that epitope 4 is the most immunogenic among the selected epitopes, suggesting that epitope display of the native conformation supports the production of a specific immune response as predicted by the in silico analysis.

Given the well-documented inherent immunogenicity of native filamentous phage particles, we also investigated the levels of phage-specific IgG antibodies in the sera of the mice used in the experiments. Notably, all single-display constructs generated high titers of phage-specific IgG antibodies, which were markedly increased after the second dose, independent of the presence or absence of the S protein epitopes displayed on the rpVIII protein (Fig. 4*B*). It is remarkable that the generation of phage-specific IgG antibodies does not appear to compromise the S protein-specific humoral response. A clear distinction was observed between epitope 4 and the other phage particles, including the negative control insertless phage. Based on these considerations, epitope 4 was therefore chosen as the lead candidate for testing a dual-display phage-based vaccine bearing a targeting moiety.

### Dual-Display Phage Construct for Pulmonary Vaccination.

To generate a dual-display phage- or AAVP-based vaccine, we optimized a simple two-step cloning strategy that allows rapid incorporation and exchange of epitopes and/or targeting peptide ligands in the phage genome. This methodology has the potential to mitigate the shortcomings of loss in vaccine efficacy due to possible mutations and to enhance the immune response by targeting phage- or AAVP-based constructs to cells or tissues. Pulmonary vaccination is among the most efficient routes to generate mucosal and systemic immunity against airborne pathogens (30–33) and has increased immunological protection of nonhuman primates infected with SARS-CoV-2 (31, 33). Thus, we prioritized CAKSMGDIVC for lung targeting and aerosol delivery (22, 23) and generated dual-display phage particles that simultaneously display epitope 4 on rpVIII (∼300 to 500 copies) and the ligand peptide CAKSMGDIVC on pIII (3 to 5 copies).

To begin to determine the immunogenic properties of the epitope 4/CAKSMGDIVC dual-display phage particles, groups of 5-wk-old BALB/c female mice were immunized with intratracheal doses. Cohorts of mice (*n* = 10 per group) received two doses of 10^9^ TU of the epitope 4/CAKSMGDIVC dual-display phage, or the epitope 4 single-display phage, or the negative control insertless phage side by side in 3-wk intervals. The presence and levels of S protein-specific IgG antibodies were evaluated in serum samples collected weekly by ELISA. Titers of S protein-specific IgG antibodies were higher in mice immunized with the epitope 4/CAKSMGDIVC dual-display phage particles compared to the control insertless phage, especially after 3 wk from the first dose, and with a substantial increase after the second dose (weeks 4 and 5) (Fig. 4*C*). These levels remained elevated for over 18 wk postimmunization with no detectable increases after another boost (*SI Appendix*, Fig. S4*A*). Epitope 4 single-display phage particles also induced systemic S protein-specific IgG antibodies but did so at levels lower than the dual-display phage particles. These results indicate that the addition of the ligand CAKSMGDIVC peptide mediates the transport of the dual-display phage from the lung airways into systemic circulation, presumably increasing the immunogenicity. As expected, dual-display phage particles also induced a strong and sustained anti-phage humoral response (*SI Appendix*, Fig. S4*B*), which firmly establishes that epitope 4/CAKSMGDIVC dual-display phage particles induce higher titers of antibody response relative to epitope 4 single-display phage particles.

Together, our findings demonstrate that epitope 4 induces a robust S protein-specific humoral response when displayed on the rpVIII protein as single-display phage particles. In addition, we demonstrate that the overall immunogenicity is enhanced when the phage are lung targeted by the CAKSMGDIVC ligand. These results establish our dual-display prototype epitope 4/CAKSMGDIVC as a translational candidate for pulmonary vaccination. They may also pave the way for admixtures of phage- or AAVP-based vaccine mixture candidates to cover multiple antigenic sites and elicit the humoral production of neutralizing antibodies with a broad spectrum of activity against the original SARS-CoV-2 strain and potentially against new variants.

### An AAVP-Based Vaccine Prototype for Gene Delivery and a Corresponding Humoral Response against the Viral S Protein.

**Fig. 5.**
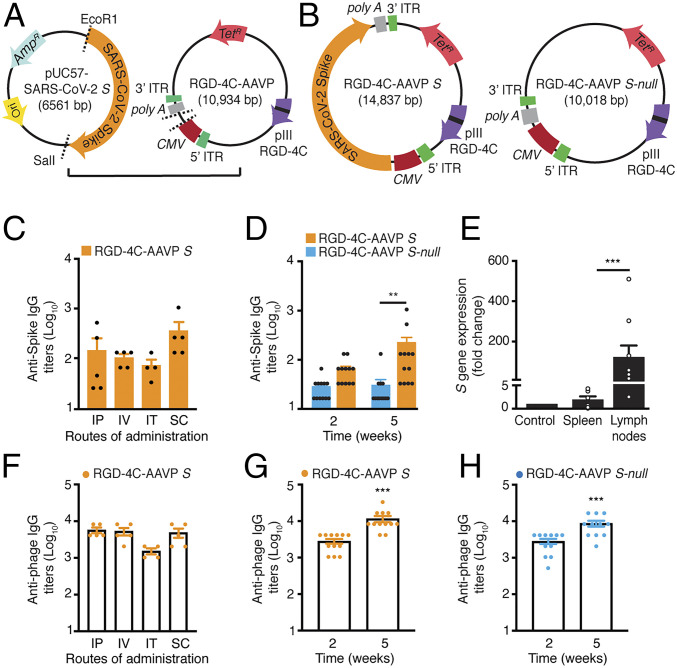
Immunogenicity of the RGD-4C-AAVP *S* in mice. Schematic representation of the AAVP-based vaccine candidate. (*A*) The modified, synthetic SARS-CoV-2 *S* protein gene was excised from the pUC57 and cloned into the RGD-4C-AAVP-TNFΔ*EcoR*I829. Expression of the *S* protein transgene cassette is driven by the constitutive *CMV* promoter and flanked by AAV ITRs. (*B*) Schematic representation of the RGD-4C-AAVP *S* and the control RGD-4C-AAVP empty vector (RGD-4C-AAVP *S-null*). (*C*) S protein-specific IgG antibody response in the sera of mice immunized with RGD-4C-AAVP *S* via different routes of administration (*n* = 5 mice per group) by ELISA. (*D*) S protein-specific IgG antibodies in sera of mice weekly immunized with RGD-4C-AAVP *S* or the control RGD-4C-AAVP *S-null* (*n* = 12 mice per group) via SC administration. Data represent ± SEM (**P < 0.01). (*E*) Tissue-specific expression of the *S* protein transgene in mice immunized with AAVP *S* 5 wk after the first immunization. Data represent ± SEM (***P < 0.001). (*F*) Phage-specific IgG antibody response in the sera of mice immunized with RGD-4C-AAVP *S* via different routes of administration (*n* = 5 mice per group). Phage-specific IgG antibody response in mice immunized with RGD-4C-AAVP *S* (*G*) or RGD-4C-AAVP *S-null* (*H*) at 2 and 5 wk after the first immunization. Phage-specific IgG antibody response was evaluated by ELISA in 96-well plates coated with 10^10^ AAVP particles per well. Tet^R^, tetracycline resistance gene. Amp^R^, ampicillin resistance gene. Ori, origin of replication.

As a parallel approach to the dual-display phage design, we also adapted a targeted AAVP vector delivery platform (20) to deliver the S protein transgene (Wuhan-Hu-1 strain, GenBank accession no. NC_045512.2), hereby designated as “AAVP *S*”. AAVP *S* displays the double-cyclic α_v_ integrin-binding RGD-4C peptide as a ligand on pIII, allowing it to bind α_v_β_3_ and/or α_v_β_5_ integrins. Such α_v_ integrins are well known to regulate the trafficking of lymphocytes and antigen-presenting cells (i.e., dendritic cells) into secondary lymphoid organs (38). For the negative control, we generated a RGD-4C-AAVP empty vector that is identical to RGD-4C-AAVP *S* but does not contain the S protein gene, hereby designated as RGD-4C-AAVP *S-null* (Fig. 5 *A* and *B*).

To assess the immunogenicity of RGD-4C-AAVP *S*, we first immunized 5-wk-old female outbred Swiss Webster mice. Cohorts of mice (*n* = 5) received 10^9^ TU of targeted RGD-4C-AAVP *S* via intraperitoneal (IP, group 1), intravenous (IV, group 2), intratracheal (IT, group 3), or subcutaneous (SC, group 4) administration routes. The S protein-specific IgG antibody response was evaluated 14 d later by ELISA (Fig. 5*C*). Baseline serum samples were used as negative controls. Administration of RGD-4C-AAVP *S* particles elicited serum IgG responses against the S protein in all experimental groups, while mice immunized via SC administration had higher serum IgG titers than the other groups. Thus, we elected to administer RGD-4C-AAVP *S* SC for subsequent in vivo assays.

We tested our immunization regimen in 5-wk-old female inbred BALB/c mice. Cohorts of mice (*n* = 12 per group) received weekly subcutaneous doses of 10^9^ TU of RGD-4C-AAVP *S* or the control, RGD-4C-AAVP *S-null*. Higher titers of S protein-specific IgG antibodies were observed in mice vaccinated with RGD-4C-AAVP *S* relative to the baseline serum samples and RGD-4C-AAVP *S-null*, particularly 5 wk after the first dose, data confirming that RGD-4C-AAVP *S* is a suitable vector for transgene delivery and elicits a systemic humoral immune response (Fig. 5*D*).

To gain insight into the S protein transgene expression mediated by RGD-4C-AAVP *S,* we investigated the fate of the targeted phage genome, namely the sites in which transduced cells were detected in mouse tissues, including main regional lymph nodes (axillary, inguinal, mesenteric, and mediastinal chains) 4 wk after the first dose. Transgene expression at varying levels was detected mainly in the draining regional lymph node chains. Skeletal muscle and spleen were used as negative control organs and showed only background levels of transgene expression (Fig. 5*E*). As predicted, transcripts for the S protein were not detected in mice immunized with the corresponding negative construct, RGD-4C-AAVP *S-null*, in side-by-side experiments. These results show that transgene delivery by targeted AAVP and the expression of the S protein in the regional draining lymph nodes trigger a systemic S protein-specific humoral response. Moreover, the data recapitulate the well-established feature of targeted AAVP particles in preventing off-site effects, even upon clearance via the reticulum-endothelial system (RES), sparing nontargeted or distal tissues, while a strong promoter drives the expression of the transgene in the transduced cells (20, 24–29). This finding is particularly noteworthy for evaluating potential adverse effects in candidate AAVP-based vaccines because off-site transduction-associated toxicities have been reported in studies of adenovirus vaccines (49, 50). Thus, the extensive body of work generated to date with targeted AAVP in cancer gene therapy (20, 24–29) could accelerate the ongoing and future design and translational development of AAVP vaccine candidates in high-throughput yet cost-effective preclinical studies.

Finally, we examined the antibody response against targeted phage in mice vaccinated with RGD-4C-AAVP *S* or RGD-4C-AAVP *S-null* phage. We observed a strong and sustained phage-specific IgG antibody response upon administration of RGD-4C-AAVP *S* by all routes of administration (Fig. 5*F*) and the response increased after boost injections in both RGD-4C-AAVP *S* (Fig. 5*G*) and RGD-4C-AAVP *S-null* (Fig. 5*H*). These results indicate that AAVP are strong immunogens likely serving as immune adjuvants for AAVP-based vaccines. Together, these results show that AAVP *S* is an enabling platform technology for delivery and expression of viral proteins, and that the targeted constructs induce a specific immune response against the S protein.

## Discussion

In this study we designed, generated, and preclinically evaluated the translational potential of phage- and AAVP-based vaccine candidates by using capsid epitope display and transgene delivery as mutually nonexclusive strategies for targeted immunization against SARS-CoV-2. We demonstrated that both experimental systems can induce an antigen-specific humoral response against the S protein and therefore represent valid candidate approaches for vaccine prototype development. Relevantly, as part of ongoing work, we have also optimized good manufacturing practice (GMP) generation, production, and purification of engineered targeted phage-based and AAVP-based particles so that industrial manufacturing could be accomplished on a large scale toward equitable access and global distribution.

One of the main challenges associated with vaccines deployed under EUA is to predict the potency and duration of the protective immune response against host cell-engaging epitopes on the S protein, particularly in the face of new genetic variant strains in the setting of a pandemic. At least in theory, focusing on structural antigen mapping and immunodominant B and T cell epitopes that trigger immune responses associated with both potent neutralizing activity and lower transmissibility would lead to long-term protection. As such, many studies have aimed to predict and/or map epitopes from B and T cells derived from the SARS-CoV-2 S protein and other structural viral proteins (51). In this study, we selected six exposed regions of the S protein with specific structural constraints for display on the phage capsid and to increase the likelihood of antigen recognition and processing by the host immune system. We found that epitope 4 (aa 662 to 671) triggered a strong and specific systemic humoral response against the S protein, presumably by recapitulating the near-native conformation of the epitope when expressed on the rpVIII protein, as indeed predicted by the molecular dynamics analyses. Of note, epitope 4 is unchanged in three main emergent SARS-CoV-2 viral lineages: Alpha, first identified in the United Kingdom (52), Beta, first identified in South Africa (53), and Gamma, first identified in Brazil (54). Thus, the combinatorial approach of selecting regions of an antigen based on conformational constraints and evaluating their structural dynamics in silico may be used to identify epitopes likely to replicate the natural immune response to a viral infection. Our findings suggest that antigen-engineering strategies, such as the case for a vaccine candidate against the Zika virus (55), have the potential to generate vaccines with high efficacy in inducing a broadly functional repertoire of neutralizing antibodies and cell-mediated immune responses.

To support the translational applications of phage-based vaccination, here we have designed an experimental protocol for immunization in mice as a proof of principle toward pulmonary vaccination against SARS-CoV-2. The design of dual-display phage particles relies on a sophisticated, yet simple bioengineering exercise: the simultaneous display of both epitope 4 on rpVIII protein and the CAKSMGDIVC targeting ligand on pIII. Because CAKSMGDIVC mediates selective targeting and transport of lung-targeted phage particles into systemic circulation (22, 23), we could employ an aerosol strategy of immunization, which may confer biological advantages over conventional routes of immunization. First, unlike SC, intramuscular (IM) or IV administration, inhalation is needle-free and minimally invasive, minimizing or eliminating the requirement for specialized medical staff, and leading to the potential for self-administration for isolated and/or vulnerable populations, particularly the elderly and immunocompromised patients. Moreover, rapid access to the upper and lower respiratory tract might lead to a reduction in pathogenic shedding of transmissible viral particles, which would ultimately result in lower rates of infection (56–58).

Antigen exposure to the lung surface, which is lined by the highly vascularized pulmonary epithelium, is a unique feature that is known to induce a local immune response that decreases infection and transmission of airborne pathogens. Recent investigational reports of intranasal and IT immunization have shown promising—if early—results against SARS-CoV-2 in mice and nonhuman primates (33, 58). Of course, optimization of targeted phage-based aerosol formulations to be delivered by suitable devices, such as portable inhalers (e.g., commercially available pressurized metered-dose inhalers, dry powder inhalers, and nebulizers), to produce particles of optimal size and mass for proper lung deposition in human patients must follow as a next logical step to advance the original vaccination platform reported here.

Finally, we have also uncovered the potential value of targeted AAVP particles to deliver the S protein gene as an alternative SARS-CoV-2 vaccine strategy. In the last decade, AAVP technology has proven to be a modular theranostic platform that may be tailored to image and treat a variety of human solid tumors in mouse models and even spontaneous tumors in domestic pet dogs (20, 24–29). These attributes make AAVP a unique platform for gene delivery. Indeed, we show that administration of RGD-4C-AAVP *S* particles elicits an antibody response in mice against the encoded S protein. Because our prototype AAVP *S* vaccine is targeted with the α_v_ integrin-binding RGD-4C peptide, which has high-affinity ligand binding for α_v_β_3_ and/or α_v_β_5_ integrins, this may facilitate targeting of inflammatory cells trafficking to the lymph nodes where gene expression and antigen presentation occur. The identification of a RGD motif within the RBD domain of the S protein suggests that integrins may act as coreceptors or an alternate path for coronavirus entry (59). Therefore, it is plausible that different functional ligands for tissue-specific transgene expression within lymph nodes (12), lymphatic vessels (60), or lung epithelial cells (e.g., CAKSMGDIVC), which has been shown to be efficient in inducing local and systemic immune response upon pulmonary delivery (22, 23), may enhance the efficacy and broad administration of targeted AAVP-based vaccines.

In conclusion, we present the experimental proof of concept and preclinical validation of the design, structure–function relationship, and initial translation of phage- and AAVP-based vaccine prototypes against COVID-19. The translational strategies introduced here—such as the targeted pulmonary vaccination through aerosol delivery and the cold-free supply chain of distribution—may become applicable against SARS-CoV-2 and other airborne transmitted infectious agents.

## Materials and Methods

### Animals.

Four-to-six-week-old Swiss Webster and BALB/c mice were purchased from The Jackson Laboratory and were housed in specific pathogen- and opportunist-free (SOPF) rooms with controlled temperature (20 ± 2 °C), humidity (50 ± 10%), light/dark cycle (light, 7:00 to 19:00; dark, 19:00 to 7:00), and access to food and water ad libitum at the research animal facilities of the Rutgers Cancer Institute of New Jersey (Newark, NJ). Littermates were randomly assigned to experimental groups. The Institutional Animal Care and Use Committee from the Rutgers Cancer Institute of New Jersey approved all animal experiments.

### Structural Analysis of S Protein for Epitope Selection.

The structure of SARS-CoV-2 S protein (PDB IDs: 6VXX, 6VYB) (4) was analyzed by using University of California, San Francisco Chimera software (61) for selection of epitopes to display on rpVIII protein. Though epitope 3 (aa 480 to 488) was not resolved in these early structures, the flanking cysteine residues of this region were predicted to form a disulfide bridge (62). This has been confirmed experimentally with since-determined structures (e.g., PDB IDs: 6ZP0, 6ZGG) (63, 64).

### Molecular Dynamics Simulations.

All-atom explicit-solvent simulations of the epitope sequences were performed with the GROMACS 2020 software package (65, 66). The initial configuration for each epitope was taken from a cryogenic electron microscopy (cryo-EM) structure of the full-length S protein (PDB ID: 6XR8) (48). Each epitope was solvated by using the TIP3P water model (67), where the box size was defined to have a 10-Å buffer between the edge of the box and the epitope. Depending on the charge of each molecule, neutralization with either Cl^−^ or Na^+^ ions was applied. The AMBER99SB-ILDN protein force field (68) was used for all simulations. After steepest-descent energy minimization, each system was equilibrated at 270 K by using the canonical (NVT) ensemble for 5 ns, followed by the isothermal-isobaric (NPT) ensemble for 5 ns, while position restraints were imposed on all heavy atoms (1,000 kJ/nm^2^). Restraints were then removed and steepest-descent minimization was performed, followed by NVT and NPT equilibration simulations (5 ns each, at 270 K). All production runs were performed in the NPT ensemble by using the Nose-Hoover thermostat (69, 70) at 310 K and the Parrinello-Rahman barostat (71) set at 1 bar. Each production simulation was performed for a minimum of 1 µs, for a total of 21 µs aggregate-simulated time. To ensure reproducibility of the results, a second set of simulations was equilibrated by using identical protocols, except that the temperature during equilibration was 310 K. The overall dynamics were not sensitive to the equilibration temperature.

### Generation of S Protein Epitopes Single-Display Phage Particles.

To generate single-display phage constructs, we used the fd-tet-derived vector f88-4 containing a recombinant gene VIII (GenBank accession no. AF218363.1). The f88-4 plasmid was digested with *Hind*III and *Pst*I restriction endonucleases and ligated with the annealed double-stranded oligonucleotides encoding for each of the six selected epitopes, as described (18, 19). Each ligation product was electroporated into electrocompetent DH5α *Escherichia coli.* Sequence-verified individual clones were used to infect K91kan *E. coli*. Phage and AAVP particles were cultured in Luria–Bertani (LB) and purified by the polyethylene glycol (PEG)-NaCl precipitation method (17). Titration of single-display phage and AAVP particles was carried out by infection of host bacterial cells K91kan *E. coli* for colony counting and represented as transducing units (TU/µL). For details, see *SI Appendix*, *Materials and Methods*.

### Generation of Dual-Display Phage Particles.

To produce phage particles simultaneously displaying SARS-CoV-2 S protein epitope 4 (aa 662 to 671) on rpVIII protein and the lung targeting peptide CAKSMGDIVC on pIII protein, we fused the single-display phage constructs (described above) and the fUSE55 genome to create a chimeric vector by double digestion of both vectors with *Xba*I and *Bam*HI restriction enzymes. Positive clones were verified by DNA sequencing analysis and the plasmid containing the chimeric vector was digested with *Sfi*I and ligated to the annealed oligonucleotides encoding the CAKSMGDIVC peptide into the fUSE55 pIII gene to generate the dual-display phage vector. The titration of dual-display phage particles was carried out by infection of host bacterial cells K91kan *E. coli*. For details, see *SI Appendix*, *Materials and Methods*.

### Genetic Engineering and Production of RGD-4C-AAVP *S* and RGD-4C-AAVP *S-Null* Particles.

The 3.821-kb SARS-CoV-2 S protein coding sequence (GenBank accession no. NC_045512.2) was synthesized at GeneWiz with modifications to simplify subcloning into the RGD-4C-AAVP-tumor necrosis factor (TNF) genome (29). The first *Eco*RI restriction site at 829 bp within the *Age*I and *Kas*I restriction sites in RGD-4C-AAVP-TNF was deleted in two steps to mutate a thymidine-to-cytosine nucleotide at position 833 without altering the translated amino acid by using the Q5 site-directed mutagenesis kit (New England Biolabs). The modified, synthetic SARS-CoV-2 S gene was ligated into the *Eco*RI/*Sal*I sites. The 84-bp transgene null sequence containing the upstream AAVP human interferon leader sequence ending with a stop codon was synthesized to produce the RGD-4C-AAVP-transgene null genome. A single transformed RGD-4C-AAVP SARS-CoV-2 *S*/MC1061 F^−^ or RGD-4C-AAVP SARS-CoV-2 *S-null*/MC1061 F^−^ colony was cultured in LB and purified by the PEG-NaCl precipitation method. For details, see *SI Appendix*, *Materials and Methods*.

### Immunization Studies in Mice.

To overcome the inherent variability associated with the immune response of the host, we tested the immunization schedule in an outbred strain (Swiss Webster) along with an inbred strain (BALB/c H-2^d^). Swiss Webster or BALB/C mice were randomized in groups of 3 to 12 animals as indicated. Group size was calculated based on statistical considerations for statistical significance. The animals were inoculated with 10^9^ TU phage particles or AAVP constructs IP, IV, IT, or SC. To evaluate long-term antibody production, we administered a third dose 21 wk following the first immunization. The devices were used to administer air-free liquid aerosol directly into the trachea of animals deeply anesthetized with 1% isoflurane (22). For the tail vein blood collections, mice were locally anesthetized with a topical solution. On day 0, blood samples were collected for the baseline, followed by consecutive blood collection every 1 to 2 wk postimmunization. Endotoxin removal was performed for each phage or AAVP preparation prior to administration of each dose, regardless of the route of administration. Phage or AAVP preparations containing endotoxin levels <0.05 EU/mL were used in this study. For details, see *SI Appendix*, *Materials and Methods*.

### Serological Analysis.

ELISAs were performed in 96-microwell plates coated with 150 ng/well of SARS-CoV-2 Spike (aa 16 to 1,213) His-tagged recombinant protein (Thermo Fisher) and 10^10^ phage or AAVP particles/50 μL of phosphate-buffered saline (PBS) overnight at 4 °C (Nunc MaxiSorp flat bottom, Thermo Fisher Scientific). Coated plates were blocked with PBS containing 5% low-fat milk and 1% bovine serum albumin (BSA) for 1 h at 37 °C. Two-fold serial dilutions (starting at 1:32) of sera in blocking buffer were added to separate the wells and incubated for 1 to 2 h at 37 °C. Following three washes with PBS and PBS containing 0.1% of Tween-20, bound antibodies were detected with an anti-mouse IgG horseradish peroxidase (HRP)-conjugated (Jackson ImmunoResearch) at optical density (OD) at 450 nm. Commercially available polyclonal IgG anti-Spike protein antibody (Thermo Fisher, MA5-35949) or anti-fd bacteriophage antibody (Sigma Aldrich, B7786) served as positive controls.

### RNA Isolation and Quantitative Real-Time PCR.

Total RNA from mice tissues was obtained with the RNeasy Mini Kit (Qiagen). First-strand cDNA synthesis was carried out with the ImProm-II Reverse Transcription System (Promega). Quantitative real-time PCR analysis was performed in a QuantStudio 5 Real-Time PCR System (Applied Biosystems). Primers and TaqMan probes were as follows: fwd 5′ GCT​TTT​CAG​CTC​TGC​ATC​GTT 3′ and rev 5′ GAC​TAG​TGG​CAA​TAA-​AAC​AAG​AAA​AAC​A 3, customized AAVP *S* 6FAM 5′ TGGGTTCTCTTGGCATGT 3-′ NFQ, Mm04277571_s1 for 18S, and Mm99999915_g1 for Gapdh. The gene expression ratio was normalized to that of 18S.

### Statistical Analysis.

Differences between groups were tested for statistical significance with Student’s *t* test or analysis of variance (one-way or two-way ANOVA) using GraphPad Prism 8. Statistical significance was set at *P* < 0.05.

## Supplementary Material

Supplementary File

## Data Availability

All study data are included in the article and/or supporting information.
