# Supplementary material for: Design and proof of concept for targeted phage-based COVID-19 vaccination strategies with a streamlined cold-free supply chain
Source: Proc Natl Acad Sci U S A. 2021 Jul 7;118(30):e2105739118. doi: 10.1073/pnas.2105739118 (PMC8325333; doi:10.1073/pnas.2105739118)
Supplement: Supplementary File [file pnas.2105739118.sapp.pdf]

PNAS
www.pnas.org

Supplementary Information for

Design and proof of concept for targeted phage-based COVID-19 vaccination strategies with a streamlined cold-free supply chain

Daniela I. Staquicini[a,b,1], Fenny H. F. Tang[a,b,1], Christopher Markosian[a,b,1], Virginia J. Yao[a,b,2], Fernanda I. Staquicini[a,b,3], Esteban Dodero-Rojas[c], Vinícius G. Contessoto[c,d], Deodate Davis[a,b], Paul O'Brien[a,b], Nazia Habib[a,b], Tracey L. Smith[a,b], Natalie Bruiners[e], Richard L. Sidman[f], Maria L. Gennaro[e], Edmund C. Lattime[g,h], Steven K. Libutti[g,h], Paul C. Whitford[i], Stephen K. Burley[g,j,k,l], José N. Onuchic[c,m,n,o,4,5], Wadih Arap[a,p,4,5], and Renata Pasqualini[a,b,4,5]

[a]Rutgers Cancer Institute of New Jersey, Newark, NJ 07101.
[b]Division of Cancer Biology, Department of Radiation Oncology, Rutgers New Jersey Medical School, Newark, NJ 07103.
[c]Center for Theoretical Biological Physics, Rice University, Houston, TX 77005.
[d]Department of Physics, Institute of Biosciences, Humanities and Exact Sciences, São Paulo State University, São José do Rio Preto, SP 15054, Brazil.
[e]Public Health Research Institute, Rutgers New Jersey Medical School, Newark, NJ 07103.
[f]Department of Neurology, Harvard Medical School, Boston, MA 02115.
[g]Rutgers Cancer Institute of New Jersey, New Brunswick, NJ 08901.
[h]Department of Surgery, Rutgers Robert Wood Johnson Medical School, New Brunswick, NJ 08901.

[i]Department of Physics and Center for Theoretical Biological Physics, Northeastern University, Boston, MA 02115.
[j]RCSB Protein Data Bank and Institute for Quantitative Biomedicine, Rutgers, The State University of New Jersey, Piscataway, NJ 08854.
[k]Department of Chemistry and Chemical Biology, Rutgers, The State University of New Jersey, Piscataway, NJ 08854.
[l]RCSB Protein Data Bank, San Diego Supercomputer Center and Skaggs School of Pharmacy & Pharmaceutical Sciences, University of California, San Diego, La Jolla, CA 92067.
[m]Department of Biosciences, Rice University, Houston, TX 77005.
[n]Department of Chemistry, Rice University, Houston, TX 77005.
[o]Department of Physics and Astronomy, Rice University, Houston, TX 77005.
[p]Division of Hematology/Oncology, Department of Medicine, Rutgers New Jersey Medical School, Newark, NJ 07103.
[1]D.I.S., F.H.F.T., and C.M. contributed equally to this work.
[2]Present address: PhageNova Bio, T. O. Daniel Research Incubator & Collaboration Center, Summit, NJ 07901.
[3]Present address: MBrace Therapeutics, T. O. Daniel Research Incubator & Collaboration Center, Summit, NJ 07901.
[4]J.N.O., W.A., and R.P. contributed equally to this work.
*To whom correspondence should be addressed. Email: jonuchic@rice.edu, wadih.arap@rutgers.edu, or renata.pasqualini@rutgers.edu

This PDF file includes:

SI Materials and Methods
Figures S1 to S4
Table S1
SI References

SI Materials and Methods

Generation of S Protein Epitopes Single-Display Phage Particles. To generate single-display phage constructs, we used the fd-tet-derived vector f88-4 containing a recombinant gene VIII (GenBank Accession Number: AF218363.1). Single colonies were selected on Luria-Bertani (LB) agar plates with tetracycline (40 µg/mL) and cultured overnight (ON). Each plasmid DNA was isolated by plasmid purification (Qiagen). Next, annealed oligonucleotides encoding for each of the six selected epitopes: epitope 1: FOR 5’AGCTTTGCCTGTCCGTTCGGCGAAGTGTTCAACGCGACCCGCTTCGCGAGCGTG TATGCGTGGAACCGCAAACGCATCAGCAACTGTCCTGCA 3’, REV 5’ GGACAGTTGCTGATGCGTTTGCGGTTCCACGCATACACGCTCGCGAAGCGGGTCG CGTTGAACACTTCGCCGAACGGACAGGCAA 3’; epitope 2: FOR 5’ AGCTTTGCCTGTTATGGCGTGAGCCCGACCAAACTGAACGATCTGTGTCCTGCA 3’, REV 5’ GGACACAGATCGTTCAGTTTGGTCGGGCTCACGCCATAACAGGCAA 3’; epitope 3: FOR 5’ AGCTTTGCCTGTAACGGCGTGGAAGGCTTCAACTGTCCTGCA 3’, REV 5’ GGACAGTTGAAGCCTTCCACGCCGTTACAGGCAA 3’; epitope 4: FOR 5’ AGCTTTGCCTGTGATATCCCGATCGGCGCGGGCATCTGTCCTGCA 3’, REV 5’ GGACAGATGCCCGCGCCGATCGGGATATCACAGGCAA 3’; epitope 5: FOR 5’ AGCTTTGCCTGTACCATGTATATCTGTGGCGATAGCACCGAATGTAG 3’, REV 5’ CAACCTGCTGCTGCAGTATGGCAGCTTCTGTCCTGCA 3’; epitope 6: FOR 5’ GGACAGAAGATCGCCACAGATATACATGGTACAGGCAA 3’, REV 5’ AGCTTTGCCTGTGTGCTGGGCCAGAGCAAACGCGTGGATTTCTGTCC 3’ were mixed at equimolar ratio and annealed using a thermocycler (93˚C for 3 min, 80˚C for 20 min, 75˚C for 20 min, 70˚C for 20 min, 65˚C for 20 min, 40˚C for 60 min). The f88-4 plasmid was digested with *Hind*III and *Pst*I restriction endonucleases and ligated with the annealed double-stranded oligonucleotides as described (1, 2). Each ligation product was electroporated into electrocompetent DH5α *E.coli.* Sequence-verified individual clones were used to infect K91kan *E.coli*. Phage and AAVP particles were cultured in LB media containing 1 mM IPTG, tetracycline (40 µg/mL), and kanamycin (100 µg/mL), and were purified by polyethylene glycol (PEG)-NaCl precipitation method (3). Titration of single-display phage and AAVP particles was carried out by infection of host bacterial cells K91kan *E. coli* for colony counting and represented as transducing units (TU/µL).

Generation of Dual-Display Phage Particles. To produce phage particles simultaneously displaying SARS-CoV-2 S protein epitope 4 (aa 662 to 671) on rpVIII protein and the lung targeting peptide CAKSMGDIVC on pIII protein, we fused the single-display phage constructs (described above) and the fUSE55 genome to create a chimeric vector. The f88-4 vector-derived DNA fragment containing the rpVIII gene was inserted into the fUSE55 phage vector by double digestion of both vectors with *Xba*I and *BamH*I restriction enzymes at 37°C, 4 h. After incubation, DNA fragments were loaded onto an agarose gel (0.8%, wt/vol). Under an ultraviolet transilluminator, the 3,925 bp fUSE55 DNA fragment and the 5,402 bp f88-4 DNA fragment containing epitope 4 in-frame with the rpVIII gene were excised. Fifty nanograms (ng) of the fUSE55 DNA fragments were ligated to 68.8 ng of the f88-4 DNA fragment with T4 DNA ligase (1U) in a final volume of 20 µL, ON at 16°C for 16 h. An aliquot of the ligation reaction was transformed into electrocompetent DH5α *E.coli* and plated onto LB agar plates containing 40 µg/mL of tetracycline. Positive clones were verified by DNA sequencing analysis and the plasmid containing the chimeric vector was purified with a QIAprep Spin Miniprep kit (Qiagen). The chimeric vector containing the f88-4 epitope 4 (aa 662 to 671) and fUSE55 was then digested with *Sfi*I at 50°C for 4 h. The digested chimera was ligated to annealed oligonucleotides encoding the CAKSMGDIVC peptide into the fUSE55 pIII gene to generate the dual-display phage vector. The titration of dual-display phage particles was carried out by infection of host bacterial cells K91kan *E.coli*.

Genetic Engineering and Production of RGD-4C-AAVP *S* and RGD-4C-AAVP *S-null* Particles. The 3,820 kb SARS-CoV-2 spike glycoprotein (*S*) coding sequence (Genbank Accession number NC_045512.2) was synthesized at GeneWiz (South Plainfield, NJ) with modifications to simplify subcloning into the RGD-4C-AAVP-TNF genome. The single *EcoR*I site in the SARS-CoV-2 S glycoprotein gene at 1378-1380 was deleted by changing the nucleotide sequence in the wobble position from "aat to aac". The corresponding asparagine, N, residue in the SARS-CoV-2 Spike glycoprotein at position 436 was unchanged. The *EcoR*I restriction site of RGD-4C-AAVP-TNF at 829 bp was deleted by substituting the thymidine nucleotide at position 833 to cytosine (Q5 mutagenesis site-directed mutagenesis kit, New England Biolabs, Ipswich, MA), which changed the translated N residue at position 200 in the tetracycline resistance gene to an aspartic acid residue. Positive RGD-4C-AAVP-TNFΔEcoRI829/MC1061 F^- clones containing a unique *EcoR*I site at 10,191 kb were verified by *EcoR*I restriction mapping

of purified dsDNA (Invitrogen PureLink HiPure Plasmid MidiPrep kit, Thermo Fisher Scientific) sequenced with overlapping sense and antisense primers by Sanger sequencing (SeqStudio, Thermo Fisher Scientific) using the BigDye Terminator v.3.1 Cycle Sequencing and XTerminator Purification kits (Applied Biosystems, Thermo Fisher Scientific) and analyzed by sequence alignment using SnapGene software (GSL Biotech, San Diego, CA). The 69-nucleotide sequence of the upstream human interferon leader sequence and the 19-nucleotide sequence of the downstream poly A region in RGD-4C-AAVP-TNF were added to the 5' or 3' ends, respectively, of the synthetic CoV-2 *S*ΔEcoRI gene to produce a 3,909 kb modified CoV-2 *S* gene that was subcloned into the *EcoR*I and *Sal*I restriction sites of pUC57/Amp^R at GeneWiz. The modified, synthetic CoV-2 *S* gene was ligated into the *EcoR*I/*Sal*I sites of dephosphorylated, gel-purified, RGD-4C-AAVPΔEcoRI829 digested with *EcoR*I-HF and *Sal*I-HF (New England Biolabs) using a 1:3 vector:insert ratio and a vector mass of 15 ng. Ligation products were transformed into animal-free, electrocompetent MC1061 F^- *E. coli* and plated onto animal-free, Lennox LB agar, pH 7.2 (US Biologicals, Salem, MA) plates containing 100 µg/mL streptomycin (VWR, Radnor, PA) and 40 µg/mL tetracycline (Gold Biotechnology, Inc., St. Louis, MO). Positive clones were verified by restriction mapping of purified dsDNA from selected transformed colonies. The 5' and 3' ends of the CoV-2 *S* gene were verified by Sanger sequencing as described above using the following primers: FOR 5' GTGGATAGCGGTTTGACTCAC 3' and REV 5' TGGTCCCAGAGACATGTATAGCATGG 3' to produce a 1,058 kb PCR product and FOR 5' AGGGCTGTTGTTCTTGTGGATCC 3' and REV 5' GGACACCTAGTCAGACAAAATGATGC 3' to produce a 268 bp PCR product, respectively. The 84 bp transgene null sequence (TGN) containing the upstream AAVP human interferon leader sequence and ends with a TGA stop codon was synthesized as a 134 bp dsDNA G-block (Integrated DNA Technologies, San Diego, CA). The synthetic TGN sequence was PCR-amplified using the following primers: FOR: 5' CGAATTGGGATCCGAGCATCG 3' and REV 5' ATCGAGGCAAGCTTCTGCAG 3', digested with *EcoR*I-HF and *Sal*I-HF (New England Biolabs), purified (Invitrogen PureLink Quick Gel Extraction and PCR Purification Combo Kit, Thermo Fisher Scientific), subcloned into dephosphorylated, gel-purified RGD-4C-AAVP-TNFΔEcoRI830 digested with the same restriction enzymes, and transformed into animal-free, electrocompetent MC1061 F^- bacteria as described above. Single transformed colonies were screened by colony PCR (DreamTaq Polymerase, Thermo

Fisher Scientific) using the following primers: FOR 5’ GTGGATAGCGGTTTGACTCAC 3’ and REV 5’ GGACACCTAGTCAGACAAAATGATGC 3’ to identify the TGN sequence as a 963 bp PCR product (Invitrogen E-gel, Thermo Fisher Scientific). Putative positive RGD-4C-AAVP-transgene null phage (AAVP *S*-null) were verified by restriction mapping and Sanger sequencing in both directions as described above. A single transformed RGD-4C AAVP *S*/MC1061 F^- or RGD-4C AAVP *S*-null/MC1061 F^- colony was used to inoculate 10 mL each of animal-free LB Broth, pH 7.4 (US Biologicals) containing 100 µg/mL streptomycin and 40 µg/mL tetracycline and grown to mid-log phase at 37 ˚C and 250 rpm in the dark. One milliliter of each mid-log phase pre-culture was used to inoculate 750 mL of animal-free LB Broth, pH 7.4 containing 100 µg/mL streptomycin and 40 µg/mL tetracycline in a sterile, 2 L shaker baffle flask for phage amplification at 30 ˚C, 250 rpm for 20 hours in the dark. Phage were precipitated twice with 15% (v/v) sterile 16.7% PEG 8000 (Sigma, St. Louis)/3.3 M NaCl (Fisher Scientific, Waltham, MA). The final phage pellet was resuspended in 1.0 mL sterile phosphate-buffered saline, pH 7.4 (Gibco, Thermo Fisher Scientific), centrifuged to remove residual bacterial debris and filter sterilized through a 0.2 µm syringe filter (MilliporeSigma, Burlington, MA). Infective phage titers (transforming units (TU)/µL) were determined by infecting K91 *E. coli* grown in animal-free terrific broth (US Biologicals) containing 100 µg kanamycin sulfate (Gold Biotechnology, Inc.) with $1x10^7$, $1x10^8$ or $1x10^9$ diluted phage, plating the infected bacteria onto animal-free LB agar plates containing 100 µg/mL kanamycin sulfate and 40 µg/mL tetracycline and counting bacterial colonies the following day. Phage genome copy number/µL was quantified by TaqMan qPCR (QuantStudio™ 7, Thermo Fisher Scientific) using the qPhage FOR 5’ TGAGGTGGTATCGGCAATGA 3’, REV 5’ GGATGCTGTATTTAGGCCGTTT 3’ primers (Invitrogen) (5) and the TaqMan® probe: 5’ VIC-TGCCGCGACAGCC-MGBNFQ (SEQ ID NO: 107) (Applied Biosystems, Thermo Fisher Scientific) using the TaqMan® Fast Advanced Master Mix (Applied Biosystems) which produced an 85 bp amplicon.

Immunization Studies in Mice

To overcome the inherent variability associated with the immune response of the host, we tested the immunization schedules in an outbred strain (Swiss Webster) along with an inbred strain (BALB/c H-2^d). Swiss Webster or BALB/C mice were randomized in groups of 3 to 12 animals as indicated. Group size was calculated based on statistical considerations for statistical significance. The animals were inoculated with 10^9 TU phage or AAVP particles IP, IV, IT or SC. For SC injections, 10^9 TU of either phage or

AAVP particles were administered with 100 µL on the front and hind limbs, and behind the neck (~ 20 µL per site). For IT vaccination, 10^9 TU of single-, dual-display phage particles or negative control insertless phage particles were administered in two serial doses in 50 µL of PBS with a MicroSprayer® Aerosolizer coupled to a high-pressure syringe (Penn-Century) and a small animal laryngoscope (Penn-Century). To evaluate long-term antibody production, we administered a third dose 21 weeks following the first immunization. The devices were used to administer air-free liquid aerosol directly into the trachea of animals deeply anesthetized with 1% isoflurane (6). For the tail vein blood collections, mice were locally anesthetized with a topic solution. On day zero, blood samples were collected for the baseline, followed by consecutive blood collection every 1-2 weeks post-immunization. Endotoxin removal was performed for each phage or AAVP particles preparation prior to administration of each dose, regardless of the route of administration. Purified phage or AAVP particles containing endotoxin was mixed with 10%Triton X-114 in endotoxin-free water, incubated on ice for 10 min, warmed to 37°C degrees for 10 min followed by separation of the Triton X-114 phase by centrifugation at 14,000 rpm for 1 min. The upper aqueous phase containing phage or AAVP was withdrawn into a sterile microcentrifuge tube. Such process was repeated from 3-5 times followed by PEG-NaCl precipitation. Resuspended phage or AAVP were sterile-filtered through 0.2 µm (Pall Corporation) syringe filters. The levels of endotoxin were measured by using the Limulus Amebocyte Lysate (LAL) Kinetic- QCL kit (Lonza). Phage or AAVP preparations containing endotoxin levels < 0.05 EU/mL were used in this study.

```
 19 TTRTQLPPAYTNSFTRGVYYPDKVFRSSV 47   164 NNCTFEYVSQPFLMDLEGK 182
      RTQLPPAYTNSFTRGVYYPDKVFRS              CTFEYVSQPFLMDLE

207 HTPINLVRDLPQGFSALEPLVDLPIGINITRFQTLLALHRSYLTPGDSSSGWTAGAAAYYVGYLQPRTFLLKYNENGTITD 287
      PINLVRDLPQGFSALEPL                                  GAAAYYVGYLQPRTFLLKYNENGTI
                  SALEPLVDLPIGINITRFQTLLALH

328 RFPNITNLCPFGEVFNATRFASVYAWNRKRISNCVADYSVLYNSASFSTFKCYGVSPTKLNDLCFTNVY 396
      PNITNLCPFGEVFNATRFAS                   YNSASFSTFKCYGVSPTK
                                              NSASFSTFKCYGVSPTKLNDLCFTN
                                                   STFKCYGVSPTKLNDLCFTN

403 RGDEVRQIAPGQTGKIADYNYKLPDDFTGCVIAWNSNNLDSKVGGNYNYLYRLFRKSNLKPFERDISTE 471
      DEVRQIAPGQTGKIADYNYKLPDDFTGCVIAWNSNNLDSKVGGNYNYLYRLFRKSNLKPFERDIS
       EVRQIAPGQTGK      KLPDD         SNNLDSKVGGN NYLYRLFRKSNLKPFERDIS
               QTGKIADYNYKLPD                          RLFRKSNLKP
                   IADYNYKLPDDFT                            SNLKPFERD

476 GSTPCNGVEGFNCYFPLQSYGFQPTNGVGYQPYRVVVLSFELLHAPAT 523
      TPCNGVEGFNC      YGFQPTNGVGYQPYRVVVLSFELLHAP
        CNGVEGFNCYFPLQSYGFQP    GYQP      SFELL

549 TGVLTESNKKFLPFQQFGRDIADTTDAVRDPQTLEILDITPCSFGGVSVITPGTNTSNQVAVLY 614
      VLTESNKKFLPFQQFGRDIA      RDPQTLEILDIT      VSVITPGTNTSNQVAV
        TESNKKFLPFQQ
        TESNKKFLPFQQFGRDIA
        TESNKKFLPFQQFGRDIA

623 AIHADQLTPTWRVYSTGSNVFQTRAGCLIGAEHVNNSYECDIPIGAGICASYQTQTNSPRRARS 686
      HADQLTPTWRVYSTGSNV            HVNNSYECDIPIGAGICA
                                          ECDIPIGAGICASYQTQTNSPRRA

738 CTMYICGDSTECSNLLLQYGSFCTQLNRALTGIAVEQDKNTQEVFAQVKQIYKTPPIKDFGGFNFSQILPDPSKPSKRSFIEDLLFNKVTLADAG 832
                 NLLLQYGSFCTQLNR   GIAVEQDKNTQEVFAQVK              NFSQILPDPSKPSKR
                              NRALTGIAVEQDKNTQEVFAQVKQIYKTPPIKDFGGFNFSQILPDPSKPSKRSFIEDLLFNKVTLA
                                ALTGIAVEQDKNTQEVFAQV QIYKTPPIKDFGGFNFSQILPDPSKPSKRSFIEDLL
                                                                           PSKPSKRSFIEDLLFNKV
                                                                           PSKPSKRSFIEDLLFNKV
                                                                             KPSKRSFIEDLLFNKVTLAD

864 LLTDEMIAQYTSALLAGTITSGWTFGAGAALQIPFAMQMAYRFNGIGVTQNVLYENQKLIANQFN 928
      TDEMIAQYTSALLAG                     MAYRFNGIGVTQNVLYENQKLIANQ

1142 QPELDSFKEELDKYFKNHTSPDVD 1165
       ELDSFKEELDKYFKNHTSPD
          SFKEELDKYFKN
           FKEELDKYFKNH
```

Fig S1. S protein epitope mapping from peer-reviewed publications. Primary sequence representation of immunogenic regions of SARS-CoV-2 S protein spanning S1 and S2 subunits identified by B-cells, T-cells, and antibody screenings on patients with COVID-19 (also listed in Table S1). Five of the six structure-guided epitopes displayed on the rpVIII protein with full- or partial-sequence overlap with other epitope mapping studies are highlighted (orange). Sequences overlapping with epitope 6 (aa 1,032 to 1,043) have not been found in previous reports.

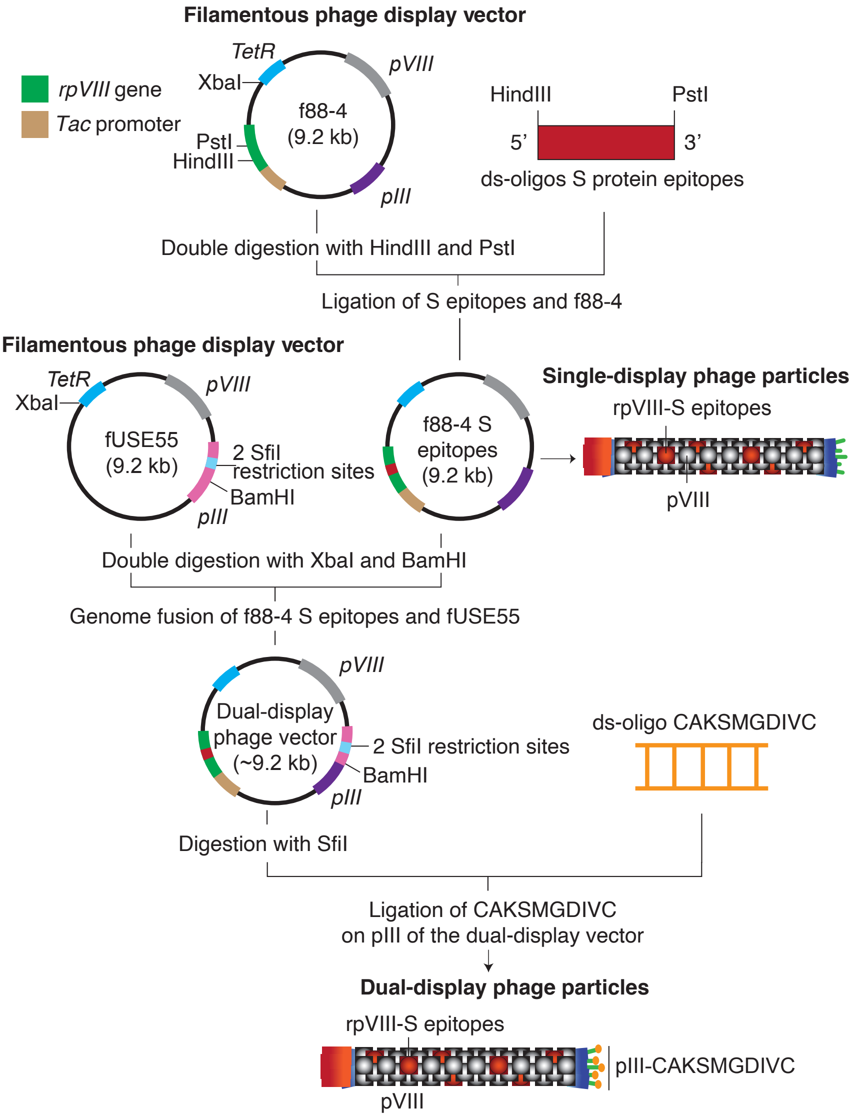

Fig S2. The single- and dual-display phage particles cloning strategy. To generate the single-display phage particles, the f88-4 phage vector was used. This vector contains two genes encoding for the major capsid protein pVIII: the wild-type (pVIII, depicted in grey) and the recombinant (rpVIII, depicted in green). rpVIII contains a foreign DNA insert between the *Hind*III and *Pst*I cloning site, which allows the cloning of annealed oligonucleotides encoding the S protein epitopes in frame with the rpVIII gene. For the dual-display phage particles, the f88-4 vector containing the epitope 4 (CDIPIGAGIC) and the fUSE55 phage vector are digested with *Bam*HI and *Xba*I restriction enzymes. The digestion products are then purified and fused according to a standard ligation protocol. The result is a chimeric vector (f88-4/fUSE55). Next, oligonucleotide encoding for the CAKSMGDIVC targeting motif was cloned within the *Sfi*I restriction site of the pIII gene (pIII, depicted in light blue), generating the dual-display phage vector, which contains epitope 4 on the rpVIII protein and the CAKSMGDIVC motif on the pIII protein. Both, single- or dual-display phage particle vectors were used to transform electrocompetent DH5α *E. coli*. Phage particles were produced in k91Kan *E. coli*. Tet^{R}, tetracycline resistance gene.

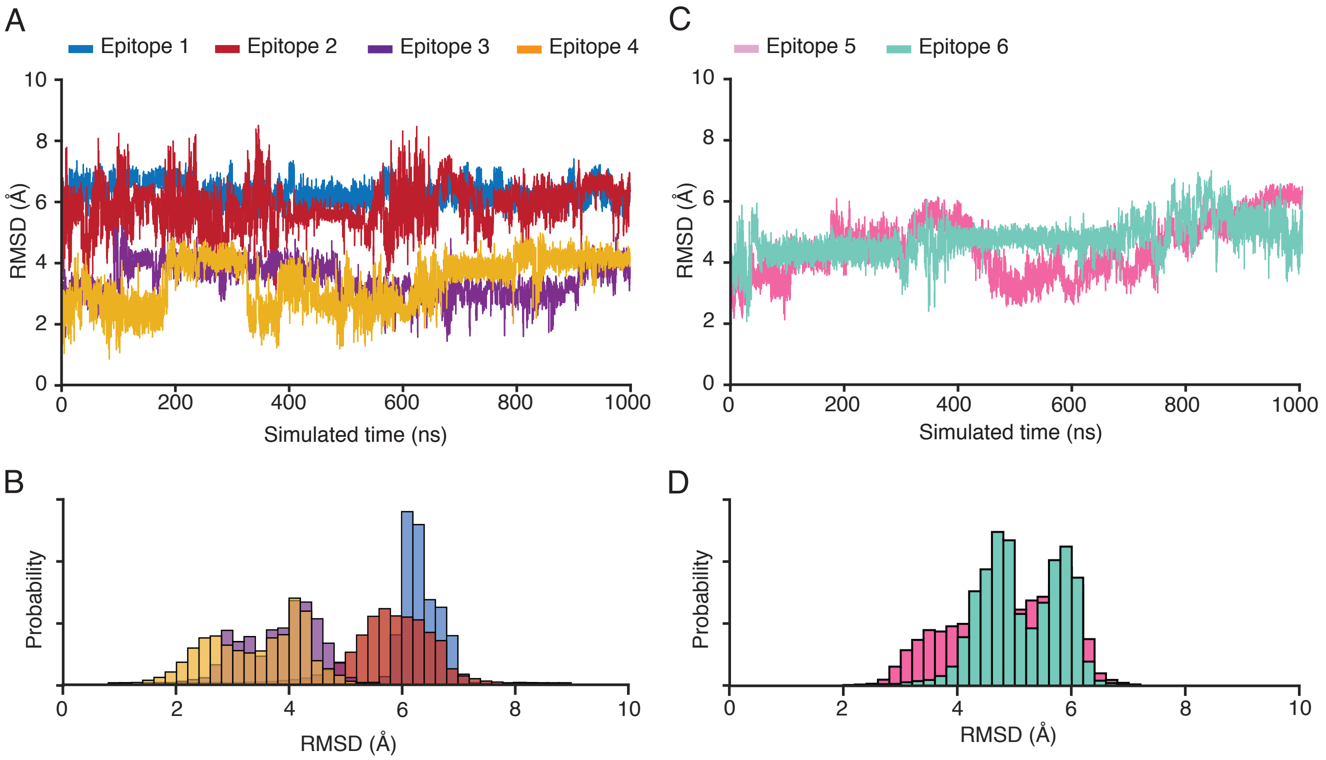

Fig S3. Second set of 1 microsecond explicit-solvent simulations of S protein epitopes. (A) rmsd from the S protein conformation for each epitope within the S1 subunit: epitope 1 (aa 336 to 361), epitope 2 (aa 379 to 391), epitope 3 (aa 480 to 488), and epitope 4 (aa 662 to 671). Epitope 4 samples the lowest rmsd (~2 Å) among all the epitopes. (B) Probability as a function of rmsd shows that epitope 4 is most likely to sample conformations similar (rmsd ~2.5 Å) to the full-length S protein conformation. (C) rmsd as a function of time for the epitopes within the S2 subunit: epitope 5 (aa 738 to 760) and epitope 6 (aa 1,032 to 1,043). Both S2 subunit epitopes maintain large rmsd values during the simulations (D).

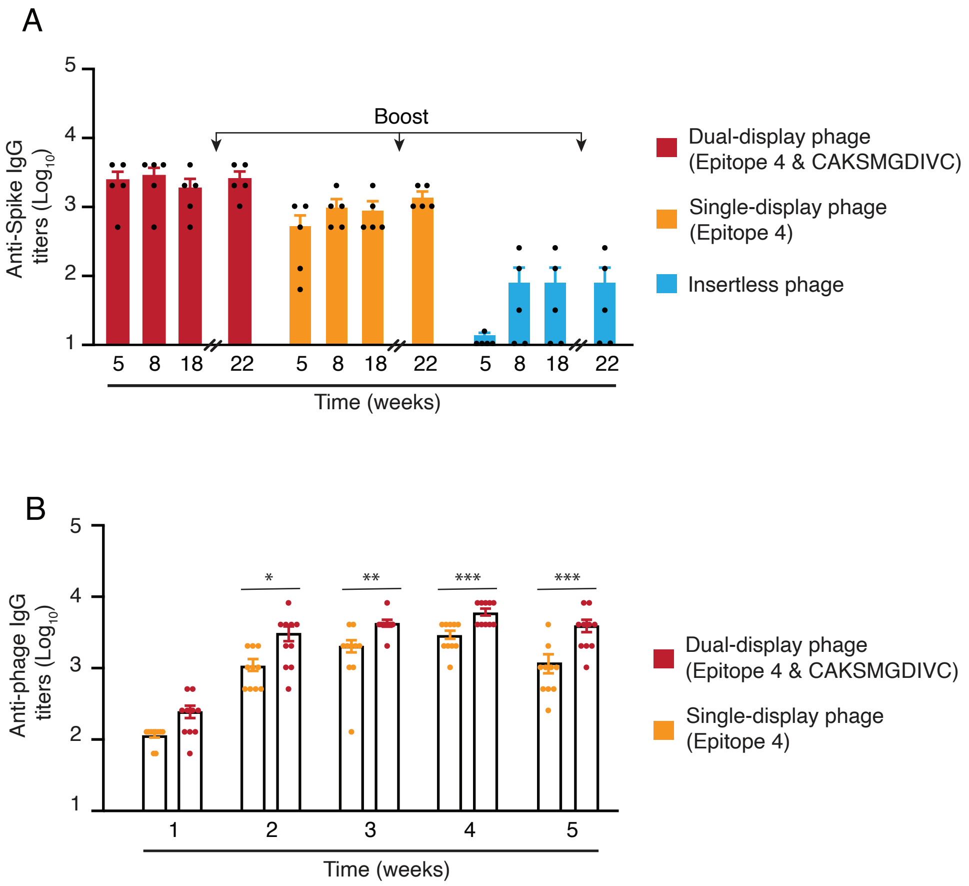

Fig S4. Immunogenicity of S protein epitopes on single- and dual-display phage particles. Five-week-old female BALB/c mice were immunized *via* IT route with 10^9 TU of epitope 4/CAKSMGDIVC dual-display phage particles, epitope 4 single-display phage particles, or the control insertless phage. (A) S protein-specific IgG antibodies were analyzed in sera of mice after 5, 8, and 18 weeks by ELISA to evaluate the long-term specific immune response (n=5 mice per group). An additional boost was administered at week 21 and the antibody response was evaluated at week 22. (B) Phage-specific IgG antibody response in the sera of mice immunized with single- and dual-display phage particles (n=10 mice per group). Data represent ± SEM (*P< 0.05, **P< 0.01, ***P< 0.001).

Table S1: Cross-reference analysis of epitope mapping of immunogenic regions of SARS-CoV-2 S protein.

Authors	Title	Residues
Amrun *et al.* (7)	Linear B-cell epitopes in the spike and nucleocapsid proteins as markers of SARS-CoV-2 exposure and disease severity	209–226<br>553–570<br>769–786<br>809–826
Poh *et al.* (8)	Two linear epitopes on the SARS-CoV-2 spike protein that elicit neutralising antibodies in COVID-19 patients	553–570<br>809–826
Farrera-Soler *et al.* (9)	Identification of immunodominant linear epitopes from SARS-CoV-2 patient plasma	655–672<br>787–822<br>1147–1158
Li *et al.* (10)	Linear epitopes of SARS-CoV-2 spike protein elicit neutralizing antibodies in COVID-19 patients	553–564<br>577–588<br>595–612<br>625–642<br>661–684<br>764–829<br>1148–1159
Noy-Parat *et al.* (11)	A panel of human neutralizing mAbs targeting SARS-CoV-2 spike at multiple epitopes	369–386
Peng *et al.* (12)	Broad and strong memory CD4+ and CD8+ T cells induced by SARS-CoV-2 in UK convalescent individuals following COVID-19	166–180<br>751–765<br>801–815<br>866–880

Shrock *et al*. (13)	Viral epitope profiling of COVID-19 patients reveals cross-reactivity and correlates of severity	406–417*<br>414–427<br>418–430*<br>424–428*<br>438–448*<br>454–463<br>459–467*<br>478–488*<br>504–507*<br>514–518*<br>551–570<br>766–785<br>811–830<br>1144–1163
Zhang *et al*. (14)	Mining of epitopes on spike protein of SARS-CoV-2 from COVID-19 patients	21–45<br>221–245<br>261–285<br>330–349<br>370–394<br>375–394<br>405–469<br>450–469<br>480–499<br>495–521<br>522–646**<br>902–926

Note: All amino acid ranges aligned to Wuhan-Hu-1 strain (GenBank Accession number: NC_045512.2).

* Extrapolated from Shrock *et al.* Fig. 7 since not explicitly stated in text.

** Not included in Fig. S1.

SI references

1. R. Rangel *et al.*, Combinatorial targeting and discovery of ligand-receptors in organelles of mammalian cells. *Nat Commun* **3**, 788 (2012).
2. R. Rangel *et al.,* Targeting mammalian organelles with internalizing phage (iPhage) libraries. *Nat Protoc* **8**, 1916-1939 (2013).
3. R. Pasqualini, E. Koivunen, E. Ruoslahti, Alpha v integrins as receptors for tumor targeting by circulating ligands*. Nat Biotechnol* **15**, 542-546 (1997).
4. M. C. Paoloni *et al*., Launching a novel preclinical infrastructure: comparative oncology trials consortium directed therapeutic targeting of TNF-alpha to cancer vasculature. *PLoS One* **4**, e4972 (2009).
5. D. I. Staquicini *et al*., Targeted phage display-based pulmonary vaccination in mice and non-human primates. *Med* **2**, 321-342.e328 (2021).
6. E. Dias-Neto *et al.*, Next-generation phage display: integrating and comparing available molecular tools to enable cost-effective high-throughput analysis. *Plos One* **4**, e8338 (2009).
7. S. N. Amrun *et al.,* Linear B-cell epitopes in the spike and nucleocapsid proteins as markers of SARS-CoV-2 exposure and disease severity. *EBioMedicine* **58**, 102911 (2020).
8. C. M. Poh *et al*., Two linear epitopes on the SARS-CoV-2 spike protein that elicit neutralising antibodies in COVID-19 patients. *Nat Commun* **11**, 2806 (2020).
9. L. Farrera-Soler *et al.,* Identification of immunodominant linear epitopes from SARS-CoV-2 patient plasma. *PLoS One* **15**, e0238089 (2020).
10. Y. Li *et al.,* Linear epitopes of SARS-CoV-2 spike protein elicit neutralizing antibodies in COVID-19 patients. *Cell Mol Immunol* **17**, 1095-1097 (2020).
11. T. Noy-Porat *et al*., A panel of human neutralizing mAbs targeting SARS-CoV-2 spike at multiple epitopes. *Nat Commun* **11**, 4303 (2020).
12. Y. Peng *et al.,* Broad and strong memory CD4(+) and CD8(+) T cells induced by SARS-CoV-2 in UK convalescent individuals following COVID-19. *Nat Immunol* **21**, 1336-1345 (2020).
13. E. Shrock *et al*., Viral epitope profiling of COVID-19 patients reveals cross-reactivity and correlates of severity. *Science* **370** eabd4250 (2020).
14. B. Z. Zhang *et al.,* Mining of epitopes on spike protein of SARS-CoV-2 from COVID-19 patients. *Cell Res* **30**, 702-704 (2020).
